# Case report: Reassessing guidelines for safe resumption of diving after spinal decompression sickness: insights from a challenging case

**DOI:** 10.3389/fmed.2024.1347465

**Published:** 2024-05-09

**Authors:** Arnaud Druelle, Jean-Eric Blatteau, Lucile Daubresse Duchadeuil, Jean Morin, Romain Roffi, Pierre-Louis Dufresne, Henri Lehot, Olivier Castagna

**Affiliations:** ^1^Ste Anne Military Hospital (HIA Ste Anne), Service de médecine hyperbare et d’expertise plongée (SMHEP), Toulon, France; ^2^LAMHESS (UPR 6312), Université de Nice, Nice, France; ^3^Underwater Research Team-ERRSO, Military Biomedical Research Institute-IRBA, Toulon, France; ^4^Military Health School of Lyon-Bron-EMSL, Bron, France

**Keywords:** decompression sickness, DCS recurrence, neurological DCS, patent foramen ovale, right-to-left shunt, spinal cord compressive factors

## Abstract

**Background:**

Recreational divers who have experienced Spinal Decompression Sickness (DCS) often aspire to return to their diving activities. Traditionally, it is recommended to observe a waiting period of several months before contemplating a return to unrestricted diving, particularly when clinical symptoms are absent, spinal cord Magnetic Resonance Imaging shows no anomalies, and the evaluation for Patent Foramen Ovale (PFO) returns negative results.

**Methods:**

This article presents a compelling case study involving a 51-year-old recreational scuba diver who encountered two episodes of spinal decompression illness within a two-year timeframe. Notably, the search for a PFO produced negative results. The primary objective of this article is to underscore the critical importance of a meticulously planned approach to resuming diving after DCS incidents, emphasizing the potential for recurrence and the essential preventive measures.

**Conclusion:**

We delve into the intricate decision-making process for returning to diving, emphasizing the significance of clinical evaluations, PFO assessments, spinal cord Magnetic Resonance Imaging, and the absence of clinical symptoms. By recognizing the risk of recurrence and the need for proactive prevention measures, we provide recommendations for both medical professionals and divers, with the ultimate goal of enhancing safety and informed decision-making within the diving community.

## Highlights

- *Resuming diving post-spinal DCS*: The study challenges the common belief that divers can safely resume diving without restrictions after surviving spinal decompression sickness (DCS) with no neurological sequelae, a clear Magnetic Resonance Imaging, and no patent foramen ovale (PFO).
- *Case study*: A compelling case report involving a 51-year-old recreational scuba diver who experienced two spinal DCS episodes within 2 years, with negative PFO results, underscores the limitations of the prevailing doctrine.
- *Prudent recommendations*: The article emphasizes the need for cautious recommendations for divers seeking to return to diving post-DCS, with a focus on clinical evaluations, PFO assessment, and the absence of clinical sequelae.
- *Risk reduction strategies*: The study suggests applying the general principles usually recommended for divers diagnosed with a low-grade Patent Foramen Ovale (PFO) to reduce risks and enhance safety for divers returning to the water post-Spinal DCS.
- *Enhanced safety and decision-making*: By addressing the recurrence risk and offering proactive prevention measures, the article aims to enhance safety and decision-making within the diving community.

## Background

Recreational underwater diving, even for leisure purposes, is undeniably a venture fraught with inherent risks (1). Despite scrupulous adherence to established protocols, particularly those governing decompression stops, the looming possibility of decompression illness remains a persistent concern (2, 3).

Among the spectrum of decompression-related incidents, spinal decompression sickness (DCS) emerges as a particularly disquieting threat, characterized by its elevated risk of neurological complications. Even when divers receive swift and appropriate hyperbaric chamber treatments, the estimated prevalence of post-incident neurological sequelae hovers around 30% (4–6).

For divers who have endured such a challenging experience and remain free of any lasting effects from this incident, two examinations are crucial for considering a possible return to diving: a spinal cord Magnetic Resonance Imaging (MRI) and the investigation of a Patent Foramen Ovale (PFO). The MRI aims to identify potential ischemic cord conditions and to determine the presence or absence of factors associated with spinal cord compression (7). The investigation of a PFO in a diver who has previously encountered a decompression accident is of paramount importance due to its potential association with severe decompression-related illnesses, particularly neurological complications (8). A PFO refers to an anomalous opening between the atria of the heart, and if it remains unsealed, it can facilitate the entry of diving gas bubbles into the circulatory system, leading to critical symptoms as they reach the brain or spinal cord. The identification of a PFO enables a more accurate risk assessment and offers the opportunity to mitigate potential complications arising from decompression sickness.

Within the domain of diving medicine, it is conventionally held that, subsequent to an episode of Spinal Cord Decompression Sickness that leaves no neurological sequelae, with no anomalies detected in the MRI, and after confirming the absence of a PFO, a diver may resume diving without any restrictions.

Nonetheless, the case study presented in this article sheds light on the limitations of this doctrine, which enjoys widespread acceptance within the diving medical community. Therefore, we advocate for the formulation of cautious recommendations for divers who have survived an episode of Spinal Cord Decompression Sickness of this nature and express an intent to return to diving. To delineate these recommendations, we propose adopting the general principles commonly advised for divers in whom a low-grade PFO has been diagnosed.

The primary objective of this article is to furnish substantial educational recommendations, underpinned by a concrete case study. Our analysis of a specific case reveals the boundaries of this prevailing doctrine and underscores the importance of introducing prudent recommendations for divers seeking to resume this activity.

## Case report

In 2017, a 51-year-old recreational diver, with a total of 100 dives, experienced his first episode of Spinal Cord Decompression Sickness. He stands at 1.72 meters, weighs 72 kg, engages in mountain biking, and is a non-smoker. He has a clean medical history and is not on any medication. Following a dive lasting 31 min in total, reaching a maximum depth of 38 meters of seawater (msw) with decompression stops (6 min at 3 msw, 3 min as per the diving computer, and an additional 3 min), he developed a partial motor deficit in his left lower extremity, which occurred 2 min after surfacing. Decompression procedures were adhered to, with no prior repetitive dives. Symptoms resolved after 30 min of normobaric oxygen therapy (NBOT). Upon admission to the hyperbaric facility at the Ste. Anne military hospital in Toulon, France, the examination showed hypoesthesia in the left leg, subtle instability during balance tests (Romberg’s test), and unsteady walking with eyes closed. No motor deficits were observed.

**Figure 1:**
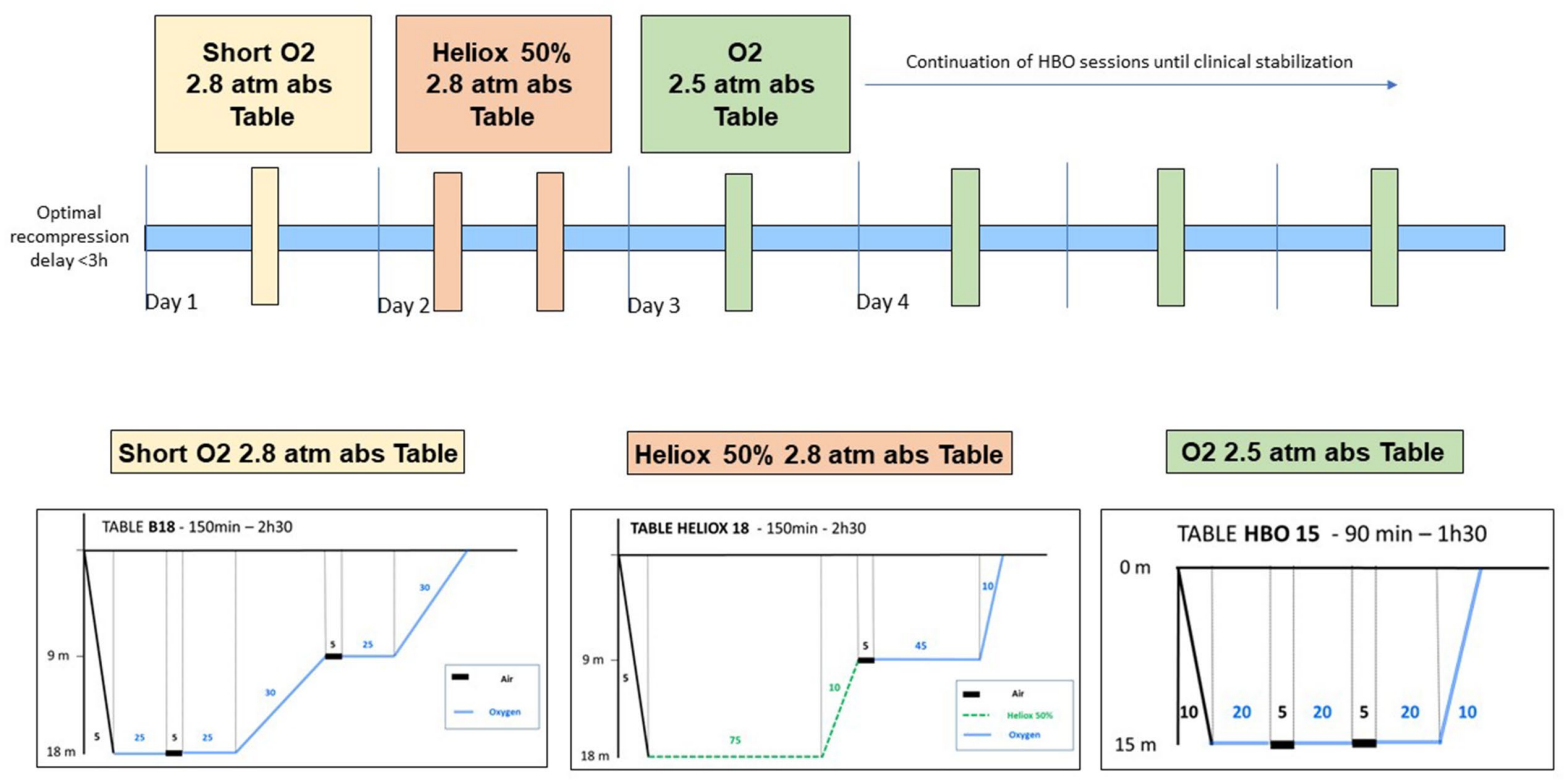
Optimal recompression treatments for severe Spinal cord DCS, as outlined in our published protocol (6).

**Figure 2:**
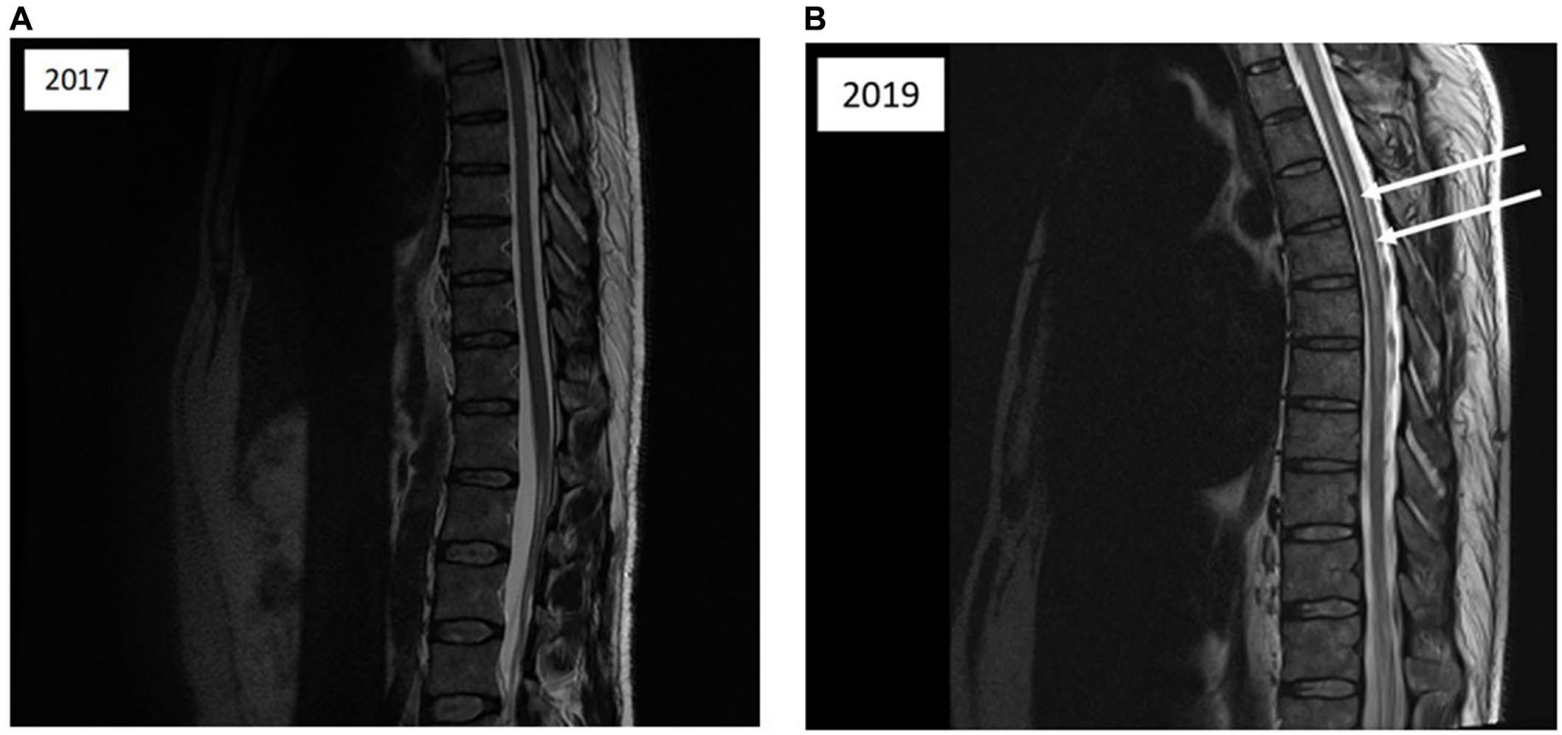
**(A)** Magnetic resonance imaging of the thoracolumbar spinal cord; sagittal T2-weighted images acquired 1 month after the initial diving incident reveal no abnormalities in spinal cord signal intensity. **(B)** Magnetic resonance imaging of the cervical spine; sagittal T2-weighted images acquired 1 month after the initial incident depict vertebral degenerative changes and medullary compression factors (indicated by arrows).

The patient underwent prompt recompression in a hyperbaric chamber. Subsequent clinical examination following hyperbaric oxygen treatment (HBOT) included recompression to 2.8 absolute atmospheres with pure oxygen for 2.5 h (Figure 1), revealed complete resolution of symptoms. The following day, the patient underwent a screening for a right-to-left shunt (RLS) using contrast-enhanced transcranial Doppler ultrasound (TCD), which produced negative results. The patient was advised to abstain from diving for 3 months. An MRI of the spinal cord was ordered to investigate potential factors contributing to spinal cord compression (Figures 2A,B). If the patient considers returning to diving, recommendations and guidance on diving limitations will be provided.

Resuming diving activities can only be considered after a thorough evaluation by a physician trained in diving medicine, which includes a comprehensive clinical assessment.

After a clinical assessment and evaluation by a diving physician affiliated with the French Diving Federation (FFESSM), the patient was granted permission to resume diving 6 months after the incident. The recovery process was incremental, beginning with pool training and progressing to depths of up to 20 meters in an inland body of water. A year following the incident, the diver completed sea dives, reaching a maximum depth of 40 meters.

In the span of 2 years, this 53-year-old diver completed 30 dives, with depths ranging from 20 to 40 meters of seawater (msw), including some consecutive dives. In 2019, during a dive lasting 43 min, he reached a maximum depth of 28 msw and followed safety decompression stops (4 min at 3 msw, as per the diving computer). The prescribed decompression procedure was meticulously adhered to, with no previous repetitive dives; his last dive was 1 month before. No contributing factors, such as unfavorable diving conditions, dehydration, or physical exertion during the dive, have been identified.

Immediately after surfacing from this dive, the patient experienced numbness in his left lower extremity. He was promptly transferred to the dive boat, where he received NBOT. After just 20 min of NBOT, he made a full recovery. Subsequently, he was evacuated by helicopter to the hyperbaric facility, where a comprehensive clinical examination revealed no abnormalities. A hyperbaric oxygen treatment (HBOT) session was conducted at 2.8 absolute atmospheres with pure oxygen for 2.5 h (Figure 1), and upon re-examination, no anomalies were detected. However, 2 h and 30 min later, the patient began experiencing motor weakness in the left lower extremity and hyperesthesia along the lateral aspect of the left thigh, descending to the mid-calf. Walking became challenging. Deep tendon reflexes remained present and normal, with no Babinski sign. In light of the worsening condition, two more recompression sessions were conducted within 24 h. These sessions involved exposure to 2.8 absolute atmospheres for 2.5 h with Heliox 50%, following our established protocol for severe DCS (Figure 1). The following day, clinical examination revealed a further decline in neurological function, marked by motor deficits in both limbs, sensory deficits in the left leg, and bilateral epileptoid tremors. Over the course of 24 h, the condition continued to deteriorate, resulting in complete flaccid paraplegia, sensory deficits encompassing tactile, epicritic, pain, and deep sensations, extending to the metameric level T6, and vesical-sphincter dysfunction.

**Figure 3:**
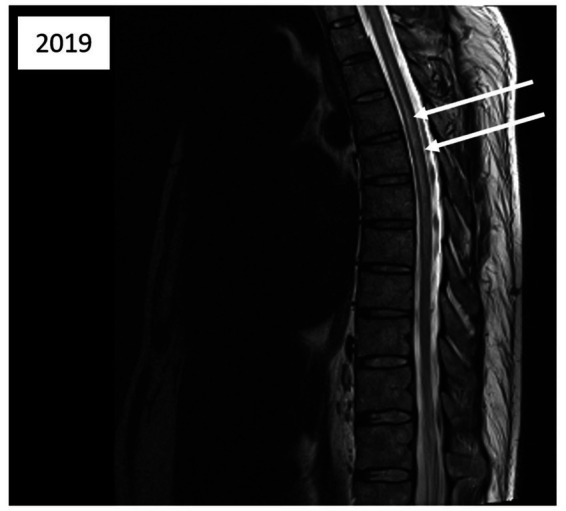
Magnetic resonance imaging of the thoracic spinal cord; sagittal T2-weighted images acquired 5 days after the second diving incident reveal abnormalities in spinal cord signal intensity (indicated by arrows).

**Figure 4:**
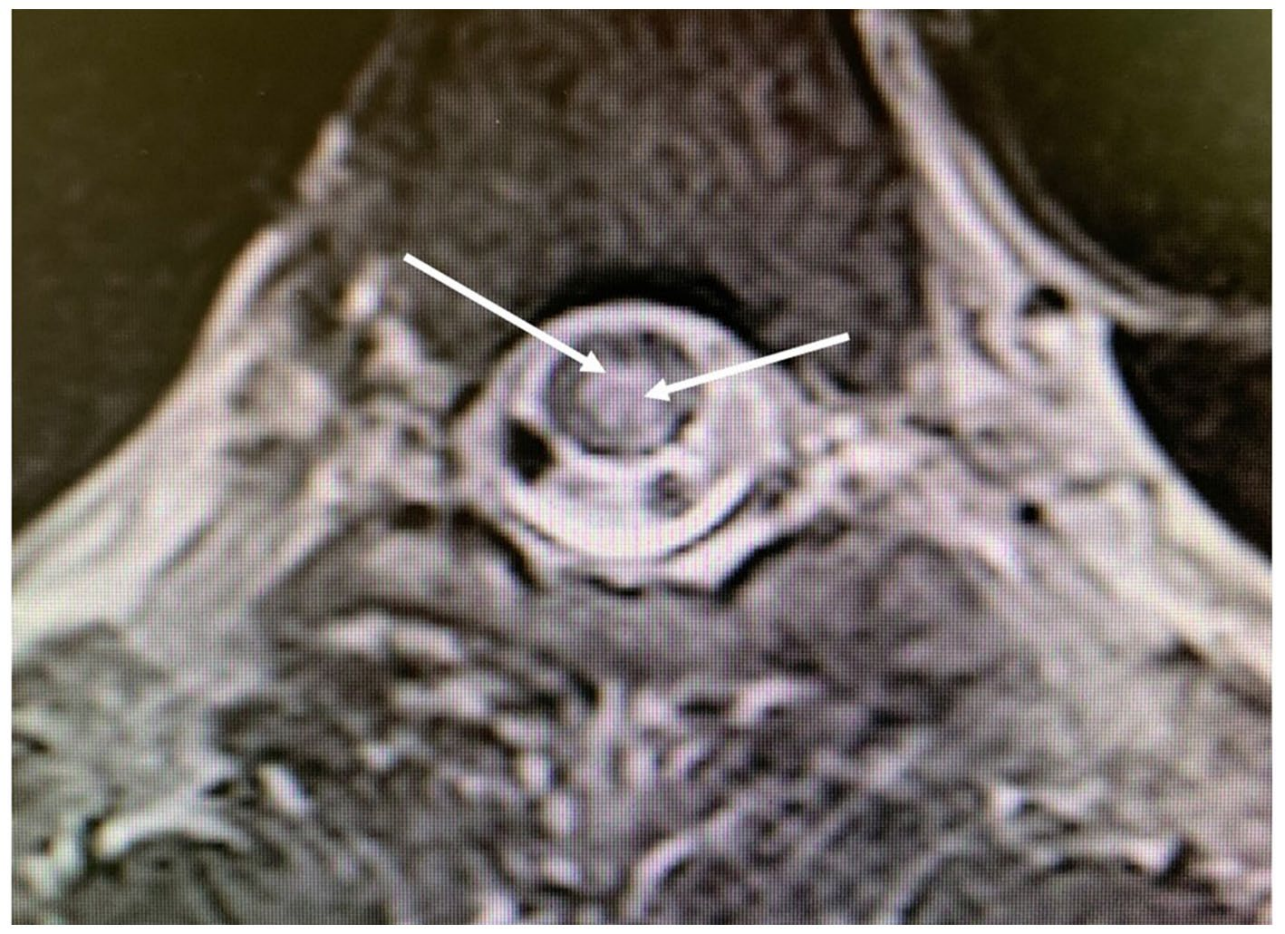
Magnetic resonance imaging of the thoracolumbar spinal cord; axial T2-weighted images obtained 5 days after the second diving incident display abnormalities in spinal cord signal intensity (indicated by arrows), with involvement of the posterior medulla and gray matter.

Five days after the second incident, a medullary MRI was performed, which revealed a T2-weighted hypersignal extending intramedullary from level T2 to T8 in sagittal sections (see Figure 3). This hypersignal affected both the posterior cords and the gray matter in the axial section (Figure 4).

Following this, an additional 12 standard HBOT sessions were administered, involving tables at 2.5 absolute atmospheres with pure oxygen for 85 min each, the patient’s condition gradually improved, although some residual motor deficits persisted in the limbs. The epileptoid tremor had subsided, but there were lingering issues with anal and vesical sphincter control. Consequently, the patient was transferred to a specialized rehabilitation facility for vascular myelopathy following hyperbaric treatment.

At this point, the patient was able to walk a distance of around one hundred meters with the assistance of a cane and required urinary catheterization multiple times a day. He still experienced fatigue during exertion. Three months after the incident, he was able to walk a few meters with the aid of crutches.

## Discussion

This clinical case represents a confirmed instance of Spinal cord DCS, with the diagnosis affirmed following a collective analysis involving several specialists in hyperbaric medicine and neurologists from our hospital. The circumstances surrounding the incident and the specific progression of symptoms were pivotal in confirming the diagnosis. No differential diagnoses of arterial dissection, neurological disease, or spinal infarction were entertained. Despite undergoing hyperbaric treatment, the unfavorable outcome illustrates a characteristic example of the “paradoxical” evolution of Spinal cord DCS, now well-recognized as the result of various immuno-inflammatory processes triggered within the first 24 h following the initial barotrauma (6). The therapeutic approach outlined in this case (see Figure 1) is deemed, in our assessment, as optimal for managing severe forms of Spinal cord DCS, in line with findings from a recent study.

This case report serves as a poignant reminder that when a diver has previously encountered spinal cord decompression sickness, displays no lingering effects after several months, and all paraclinical examinations yield normal results, the potential for a recurrence of spinal DCS cannot be entirely dismissed. We intend to revisit this case report to thoroughly analyze the insights it may offer for improving the management of divers following such accidents, with the aim of minimizing the risk of recurrence to the greatest extent possible.

At the hyperbaric facility of Ste. Anne Hospital, Neurological decompression sickness stands as the predominant DCS variant, accounting for roughly 40–45% of all DCS admissions. Throughout their hospital stay, patients at the highest risk of developing post-DCS complications undergo systematic evaluation through spinal cord MRI scans to identify potential ischemic cord conditions. Additionally, MRI can be employed to assess the presence or absence of factors related to spinal cord compression, which appears to be a risk factor contributing to the severity and likelihood of DCS recurrence (7, 9, 10). The systematic search for RLS using TCD is a standard procedure for any case of neurological DCS. In instances where a significant RLS is detected, transesophageal echocardiography is carried out to confirm the presence and morphology of the PFO (8).

Medical literature has extensively described the relationship between the presence of PFO and DCS (11). To reduce the incidence of neurological injury, closure of the PFO has been suggested (12). However, the issue of PFO should not overshadow the search for other risk factors associated with the occurrence and recurrence of DCS, such as the presence of medullary compression factors, as demonstrated by spinal cord MRI (4, 13, 14).

Following a 3-to-6-month interval, the assessment for a return to diving should be conducted, and approval may be granted, contingent on the absence of clinical or paraclinical aftereffects. In situations where residual sequelae persist, the physician must carefully evaluate the nature and severity of these sequelae, consider the diving type (e.g., recreational, non-decompression), and take into account the diver’s awareness of the associated risks before allowing a return to diving. It’s imperative to ensure that any resumption of diving is preceded by precautionary measures aimed at minimizing the risk of bubble formation during decompression (refer to “Recommendations section”).

In this particular case, an MRI scan to identify spinal cord compression factors was conducted following the initial episode of DCS. The results revealed the presence of small, staggered protrusions at the cervical spine level (Figure 2B). However, the degree of compressive impact on the spinal cord does not seem to be substantial enough to warrant a restriction on resuming diving after the initial DCS incident. We propose that the hyperbaric physician should take into consideration anatomical compression, whereas typically, radiologists tend to emphasize only surgical indications of medullary compression, which involve the loss of safety white matter.

In the case presented, there are indeed small areas of disc protrusion on the spinal side at the cervical level (see Fig), but they are minimal. Following discussions with radiologists and neurologists, these anomalies were not deemed significant enough to directly impact the spinal cord or the venous drainage circulation of the spinal cord, which could explain the observed impairment at the dorsal level.

Moreover, the search for a RLS yielded negative results. Numerous studies have documented the incidence and recurrence of DCS in divers with RLS or PFO, with a higher likelihood when the PFO exhibits high permeability (11, 14). It’s essential to acknowledge that the presence of a PFO in cases of medullary DCS is less common, with approximately 50% exhibiting a PFO, compared to cerebral, cochlear-vestibular, or cutaneous DCS where the prevalence of PFO is around 80%. Therefore, the absence of RLS or PFO in this patient does not come as a surprise.

After experiencing the initial episode of DCS, the diver eventually received a positive medical assessment permitting a return to diving following a three-month period of recuperation. It was emphasized, following hyperbaric treatment, that strict adherence to diving restrictions was paramount. However, regrettably, there has been no modification in the diver’s diving practices. The primary factors that appear to account for the recurrence of this DCS are non-compliance with the diving recommendations and the prior history of neurological DCS.

From a pathophysiological perspective, medullary DCS remains a subject of incomplete comprehension. Three mechanisms are consistently cited as potential factors:

- *In-situ* generation of decompression bubbles within neurological tissues.
- Venous stasis within the medullary drainage circulation.
- Arterial embolization of the medullary circulation due to RLS.

In this case report, the spinal cord compression observed in the cervical MRI, conducted after the initial spinal cord DCS, was deemed anatomically insignificant and appeared unlikely to impede venous medullary circulation.

Another aspect for discussion pertains to the region of spinal cord injury identified during the MRI conducted after the second incident. Literature data from several years ago (7, 9) indicate that MRI findings in cases of Spinal cord DCS more commonly reveal ischemic lesions in the posterior or lateral columns of the spinal white matter, with central gray matter involvement being less prevalent. Nevertheless, gray matter involvement can also manifest in certain severe cases of Spinal cord DCS (15), as observed in the case we present (Figures 3, 4). We attribute this observation to advancements in imaging techniques with newer generations of MRI, facilitating a more precise analysis of spinal cord lesions, including the more frequent detection of gray matter involvement in Spinal cord DCS cases compared to previous methodologies.

Nonetheless, the recurrence of spinal cord DCS, with symptoms akin to the initial episode, hints at the possibility of parallel neurological tissue damage at the cervical level. The absence of visible sequelae after the first occurrence, both clinically and upon paraclinical assessment (spinal cord MRI), does not necessarily imply the absence of subclinical lesions. It’s plausible that contemporary imaging techniques may not have the capability to detect these subtle abnormalities. Given that patients with DCS often have a history of prior incidents, we posit that neurological tissue damage during the initial DCS episode may, in itself, serve as a risk factor for recurrence (4, 7, 16).

## Limitations

Although this article focuses on a single case study, which inherently presents challenges in extrapolating generalized conclusions, it is crucial to emphasize the educational aspect of our work. While our findings may have limited applicability to broader populations, they serve as a poignant reminder of the complexities inherent in determining a diver’s suitability to return to diving after experiencing neurologic decompression sickness, even in the absence of apparent sequelae. By spotlighting this case, our objective is not only to inform and educate divers but also to raise awareness among the diving medical community regarding the nuanced considerations involved in such decisions. Therefore, while acknowledging the inherent limitations, we believe this manuscript holds significant value in its ability to contribute to the ongoing discourse on diver safety and medical decision-making.

## Recommendations

In the realm of diving medicine, it is a conventional belief that divers who have experienced DCS without enduring neurological consequences, exhibiting clear MRI scans, and confirming the absence of a PFO, can safely resume their diving activities without restrictions.

However, the case study featured in this article casts a spotlight on the constraints of this widely embraced doctrine within the diving medical community. Thus, we advocate for the formulation of prudent recommendations aimed at divers who have overcome such an episode of Spinal DCS and express a desire to reengage in diving. In defining these guidelines, we suggest drawing from the overarching principles routinely recommended for divers diagnosed with a low-grade PFO. These recommendations seem pertinent for preventing recurrence and are applicable even to divers who do not exhibit a right-to-left shunt, as in the case presented.

These recommendations can be grouped into three categories:

1. *To reduce bubble formation during decompression:*

- Limit dive depth to no more than 30 meters of seawater (msw),
- Restrict the duration of dives,
- Avoid dives that require decompression stops,
- Refrain from repetitive dives,
- Maintain a consistent depth profile during the dive,
- Prefer Nitrox diving,
- Use Nitrox or pure oxygen during decompression stops,
- Adhere to a gradual progression in both dive depth and duration, especially for multi-day activities,
- Minimize significant physical exertion during the dive.

1. *To reduce the risk of right-to-left shunting:*

- Avoid forced Valsalva maneuvers while diving,
- Refrain from reboarding with a scuba tank secured to your back,
- Avoid strenuous physical effort within 6 hours after a dive,
- Do not engage in breath-hold diving immediately after scuba diving,

1. *To ensure medical fitness:*

- After the age of 45, consider limiting diving activity,
- Maintain good physical fitness, favoring endurance exercises,
- Engage in regular and progressive diving training,
- Limit diving activity in cases of overweight or sedentary lifestyle,
- Discontinue diving in the presence of concurrent illnesses or treatments,
- Abstain from diving in the wake of recent emotional stress,
- Avoid diving when fatigued; do not dive after a short or alcohol-infused night,
- Consume isotonic beverages regularly before diving.

## Conclusion

Resuming diving after Spinal Decompression Sickness should be a decision based on both clinical and radiological assessment.

The abundance of literature on PFO and DCS recurrence should not make us forget that the absence of PFO is not a “protective factor” for future diving.

It is also important to look for spinal cord compression factors (MRI) and to assess the individual risk of recurrence. Interpretation of this imaging study can be sensitive and requires expert opinion to determine whether or not the abnormality is compressive.

In any case, a diver with a history of DCS should be aware of the environmental conditions that promote bubble formation and should minimize this risk by keeping their dive profiles as safe as possible. We find it unreasonable to permit their return to diving without the application of restrictions, as is the current practice.

Diving with nitrox by itself does not reduce DCS risk unless diving on nitrox but adhering to air tables, especially if diving to the edge of no-decompression limits.

After identifying and taking into account all contributing factors, it is imperative for the clinician to engage in a dialogue with the injured diver to ascertain their resolve to return to diving activities. If the determination to resume diving remains steadfast, the implementation of diving restrictions represents the most effective strategy for preventing the recurrence of DCS.

We advocate for additional testing to assess risk factors promptly following the first incident, along with the provision of recommendations aimed at preventing recurrences.

## Data availability statement

The original contributions presented in the study are included in the article/supplementary material, further inquiries can be directed to the corresponding authors.

## Ethics statement

Written informed consent was obtained from the individual(s) for the publication of any potentially identifiable images or data included in this article.

## Author contributions

AD: Conceptualization, Investigation, Writing – original draft, Writing – review & editing. J-EB: Writing – original draft. LD: Investigation, Writing – review & editing. JM: Writing – review & editing. RR: Writing – review & editing. P-LD: Writing – review & editing. HL: Writing – review & editing. OC: Writing – original draft, Data curation, Formal analysis, Validation, Visualization, Writing – review & editing.
